# Health-Related Quality of Life in Pediatric Hepatic Glycogen Storage Disease: A Dual-Perspective Study

**DOI:** 10.3390/nu18132099

**Published:** 2026-06-27

**Authors:** Oznur Aydin, Selcuk Dagdelen, Hasan Ozen, Hulya Gokmen-Ozel

**Affiliations:** 1 Department of Nutrition and Dietetics, Faculty of Health Sciences, Hacettepe University, Ankara 06100, Türkiye; oznuraydin@hacettepe.edu.tr; 2 Division of Endocrinology and Metabolism, Department of Internal Medicine, Faculty of Medicine, Hacettepe University, Ankara 06230, Türkiye; dagdelens@hacettepe.edu.tr; 3 Division of Pediatrics, Department of Internal Medicine, Faculty of Medicine, Hacettepe University, Ankara 06230, Türkiye; haozen@hacettepe.edu.tr

**Keywords:** pediatric quality of life inventory, uncooked cornstarch, nutritional management, body composition, parent-proxy report

## Abstract

**Background/Objectives**: Hepatic glycogen storage diseases (GSDs) are rare inherited metabolic disorders requiring lifelong nutritional management and strict metabolic control, which may adversely affect the health-related quality of life (HRQoL). This study aimed to evaluate the HRQoL in children with hepatic GSD using both child and parent reports, compare findings with normative data, and explore associations with biochemical, anthropometric, and nutritional management-related parameters. **Methods**: The study included 23 children with hepatic GSD and their parents. HRQoL was assessed using the Pediatric Quality of Life Inventory (PedsQL) Generic Core Scale. Child and parent reports were compared with normative data for healthy and chronically ill children. Agreement between child and parent reports was evaluated with intraclass correlation coefficients, and exploratory associations between variables were assessed using partial Spearman correlation analyses. **Results**: Total and most subscale PedsQL scores reported by both children and parents were significantly lower than those of healthy peers and children with chronic diseases (*p* < 0.05). Parents reported lower HRQoL scores than children, particularly in psychosocial, social, and school functioning domains, with low to moderate agreement between reports. Exploratory analyses suggested that body composition and nutritional treatment burden indicators were correlated with selected HRQoL domains. **Conclusions**: Children with hepatic GSD experience impaired HRQoL from both child and parent perspectives. Integrating HRQoL assessment into routine clinical and nutritional follow-up may help identify unmet psychosocial and dietary support needs and support more individualized nutritional management in children with hepatic GSD.

## 1. Introduction

Glycogen storage diseases (GSDs) are rare inherited metabolic disorders caused by defects in enzymes or regulatory proteins involved in glycogen metabolism [1]. GSDs are mainly classified as hepatic or muscular forms, and hepatic types (0, I, III, VI, IX, and Fanconi–Bickel syndrome) may present with hypoglycemia, hyperlipidemia, hyperlactatemia, hyperuricemia, and elevated liver enzymes [1,2,3]. The management of hepatic GSDs requires a multidisciplinary approach tailored to subtype-specific needs, aiming to control hypoglycemia, prevent long-term complications, and enhance health-related quality of life (HRQoL) [3]. This includes uncooked cornstarch (UCCS) use and frequent feedings to prevent hypoglycemia, pharmacological therapies for complication management, and closely monitored follow-up [4,5]. Therefore, the management of these diseases can be extremely challenging for patients and their families [6,7].

In pediatric populations, chronic diseases may restrict children’s participation in daily life and social activities, potentially compromising their HRQoL [8,9,10,11]. This issue is also increasingly recognized in hepatic GSD, for which a recent international priority-setting partnership identified comprehensive investigation of HRQoL as a research priority [6,7,12,13]. However, published evidence on HRQoL in children with hepatic GSD remains very limited, with only a few small-scale studies available to date [7,12]. A study involving 31 children and adolescents with GSD type I showed that the HRQoL of patients was similar to that of children with chronic diseases, but significantly lower than that of their healthy peers [7]. In another study carried out on 52 children with GSD, it was shown that GSD patients scored significantly lower quality of life scores than their healthy peers [12].

Although self-report is considered the preferred approach for assessing HRQoL, factors such as age, developmental level, cognitive capacity, and language skills should be considered in pediatric populations [14,15]. Therefore, understanding the level of agreement between child self-reports and their parent-proxy reports is crucial for accurate assessment [14]. Various studies on children with chronic diseases have shown that parents generally perceive the impact of the disease as more severe than their children do and evaluate HRQoL lower [16,17]. But the number of studies examining both children’s and parents’ perspectives of HRQoL in children with GSD is quite limited [7].

Thus, the objectives of this study are (1) to evaluate the HRQoL of pediatric hepatic GSD patients from the perspective of both children and parents and to compare these with normative data from healthy children and children with other chronic diseases, (2) to determine the consistency between child self-reports and parent-proxy reports, and (3) to explore nutritional management-related indicators, body composition, and biochemical variables associated with HRQoL in children with hepatic GSD.

## 2. Materials and Methods

### 2.1. Study Design and Participants

This cross-sectional study included 23 patients aged 3–18 years with confirmed hepatic GSD types I, III, IV, VI, IX, or Fanconi–Bickel syndrome, who were followed at the Division of Gastroenterology, Hepatology, and Nutrition, Hacettepe University Faculty of Medicine, between May 2024 and November 2025.

### 2.2. Preliminary Questionnaire

A structured preliminary questionnaire was used to collect sociodemographic data, including participants’ age and sex, maternal age and education level, primary caregiver, and parental consanguinity. Nutritional management-related indicators, including daily meal frequency, maximum fasting tolerance, hypoglycemia frequency, and UCCS use, were assessed based on patient and/or caregiver self-report.

### 2.3. Biochemical Analysis

Fasting blood glucose, lactate, uric acid, liver transaminases [aspartate aminotransferase (AST) and alanine aminotransferase (ALT)], and lipid profile data were obtained from the hospital system based on morning fasting blood samples collected during routine follow-up visits, considering individual fasting tolerance.

### 2.4. Anthropometric Measurements

Height and weight were measured using a calibrated stadiometer and digital scale, respectively, and BMI was calculated as weight divided by height squared (kg/m^2^). Age- and sex-specific height and BMI z-scores were determined using WHO references [18]. The body composition of 20 patients aged 5 and older (due to the device’s technical limitations) was evaluated using the bioelectrical impedance method (TANITA MC-980MA-N plus, Tanita Corporation, Tokyo, Japan) [19].

### 2.5. Assessment of Health-Related Quality of Life

To assess participants’ HRQoL, the Pediatric Quality of Life Inventory (PedsQL) 4.0 Generic Core Scales, developed by Varni et al. [20], were used. The PedsQL includes age-specific forms; only a parent-proxy form is available for children aged 2–4 years, whereas both child self-report and parent-proxy forms are available for ages 5–18 years. The Turkish validity and reliability of the scale have been established for all age groups [21,22,23]. Accordingly, only parent-proxy reports were obtained for children aged 2–4 years (*n* = 3), while both child self-reports and parent-proxy reports were obtained for children aged 5–18 years (*n* = 20). All parent-proxy forms were completed by the children’s mothers, who were their primary caregivers.

The scale is scored using a Likert-type assessment according to the PedsQL scoring algorithm [24], with responses ranging from “never a problem” (0) to “always a problem” (4), then reverse-scored and transformed to a 0–100 scale. The scale includes emotional, social, school, and physical functioning domains. In the 2–4 year age group, school functioning items were scored only for children attending nursery or preschool, resulting in two missing parent-proxy values for this subscale. Psychosocial health is calculated from the emotional, social, and school functioning domains, and the total score is calculated by averaging all items. Higher scores indicate better HRQoL [24].

To provide a reference framework for interpreting HRQoL scores, child self-report and parent-proxy PedsQL scores were compared with reference values previously reported by Varni et al. [25], which were derived from healthy children and pediatric patients with chronic diseases in the United States. This dataset was selected because it includes both healthy and chronically ill pediatric populations across a comparable age range and represents one of the most widely used references for interpreting PedsQL Generic Core Scale scores [25]. Although the Turkish version of the PedsQL has been validated for different age groups, comprehensive Turkish normative data covering all relevant age groups and comparable clinical reference groups were not available. Therefore, these data were used as a reference framework for contextualizing HRQoL scores, rather than as a culturally matched control group [25].

### 2.6. Statistical Analysis

Participants’ sociodemographic, anthropometric, and biochemical characteristics were summarized using descriptive statistics. Normality was assessed visually using histograms and Q-Q plots and analytically using the Shapiro–Wilk test. Given the limited sample size and the non-normal distribution of most variables, non-parametric tests were used. Continuous variables are presented as mean ± standard deviation (SD) for comparisons with normative data and otherwise as median and interquartile range (IQR), while categorical variables are presented as frequency and percentage. Given the limited number of patients within individual GSD subtypes, primary analyses were conducted in the overall cohort, and exploratory comparisons between patients with GSD type I and other hepatic GSD subtypes are presented in Supplementary Table S1. All analyses were performed using IBM SPSS Statistics for Windows, Version 23.0 (IBM Corp., Armonk, NY, USA), and a two-tailed *p*-value < 0.05 was considered statistically significant.

Internal consistency was assessed using Cronbach’s alpha coefficients, which were ≥0.78 across all domains for both child and parent PedsQL forms. Total score Cronbach’s alpha values were 0.80 for child self-reports and 0.83 for parent-proxy reports, supporting the reliability of the PedsQL in both respondent groups [26]. PedsQL scores from child self-reports and parent-proxy reports were compared with normative data [25]. Since raw normative data were not available, one-sample *t*-tests were performed using the reported mean, SD, and sample size, and 95% confidence intervals were calculated. Cohen’s d was used to assess effect size, with values of 0.20–0.49, 0.50–0.79, and ≥0.80 interpreted as small, medium, and large effects, respectively [27].

Differences between child self-report and parent-proxy PedsQL scores were assessed using the Wilcoxon signed-rank test, and effect sizes were calculated using the rank biserial correlation coefficient (r), interpreted as very small (<0.10), small (0.10–0.29), medium (0.30–0.49), and large (≥0.50) [28]. Additionally, agreement between child and parent reports was evaluated using intraclass correlation coefficients (ICCs), classified as poor (≤0.40), moderate (0.41–0.60), substantial (0.61–0.80), and almost perfect (≥0.81) [29].

Exploratory partial Spearman correlation analyses adjusted for age and sex were performed to assess associations between PedsQL scores and anthropometric, biochemical, and nutritional variables. Hypoglycemia frequency, categorized as none, rare (≤1/month), and frequent (≥1/week), was also included in the correlation analyses. Correlations were visualized as a heatmap.

## 3. Results

The general characteristics of the children and their mothers are presented in Table 1. The median age of the children was 13.00 years, and 65.2% were male. Overall, 87.0% were diagnosed with GSD type I, III, or IX. Patients with GSD type IV, GSD type VI, and Fanconi–Bickel syndrome represented single cases. Available subtype-level information included type Ia (*n* = 3), type Ib (*n* = 2), type IIIa (*n* = 6), type IIIb (*n* = 1), type IXa (*n* = 2), and type IXc (*n* = 1). Most patients used UCCS (*n* = 18, 78.3%), while five (21.7%) did not; among non-users, three had GSD type III, one type IV, and one type IX. Under current dietary management, 69.6% reported no hypoglycemia, whereas 21.7% experienced rare episodes (≤1/month) and 8.7% frequent episodes (≥1/week). No severe hypoglycemic episodes requiring hospital-based intervention were reported; reported episodes were mild to moderate according to patient- and/or caregiver-reported information. The median age of primary caregivers was 38.00 years, and 73.9% had at least a secondary school education.

Anthropometric, biochemical, and nutritional characteristics of the participants are summarized in Table 2. The median height-for-age z-score was −1.52, indicating a tendency toward short stature, while the median BMI-for-age z-score and body fat percentage were 0.27 and 26.2%, respectively. Biochemical parameters showed considerable interindividual variability. To improve the clinical clarity of the findings, the number and percentage of patients with values outside the reference range for each biochemical parameter are also presented in Table 2. The most frequent elevated biochemical findings were ALT (*n* = 18, 78.3%), AST and lactate (*n* = 17, 73.9% each), triglycerides (*n* = 16, 69.6%), and total cholesterol (*n* = 13, 56.5%). The median daily meal frequency was 7, and self-reported maximum fasting tolerance ranged from 4 to 10 h. Among UCCS users, the median intake frequency was twice daily, with a median total intake of 48 g/day, corresponding to 2.09 g/kg body weight/day.

Table 3 presents the mean PedsQL scores for child self- and parent-proxy reports along with comparisons to normative data from healthy and chronically ill children. Mean values for the total score and most PedsQL subscales were significantly lower in children with GSD than in both chronically ill and healthy peers (*p* < 0.05). However, no significant differences were observed for the emotional and social functioning subscales in child self-reports (*p* > 0.05).

Median and IQR values for child self-reported and parent-proxy PedsQL scores are presented in Table 4. Parents reported significantly lower total, psychosocial health, social functioning, and school functioning scores than children (*p* < 0.05), with medium-to-large effect sizes, whereas physical health and emotional functioning scores did not differ significantly (*p* > 0.05). ICC analyses indicated poor-to-moderate agreement between child and parent-proxy reports of health-related quality of life (ICC = 0.023–0.451).

**Figure 1:**
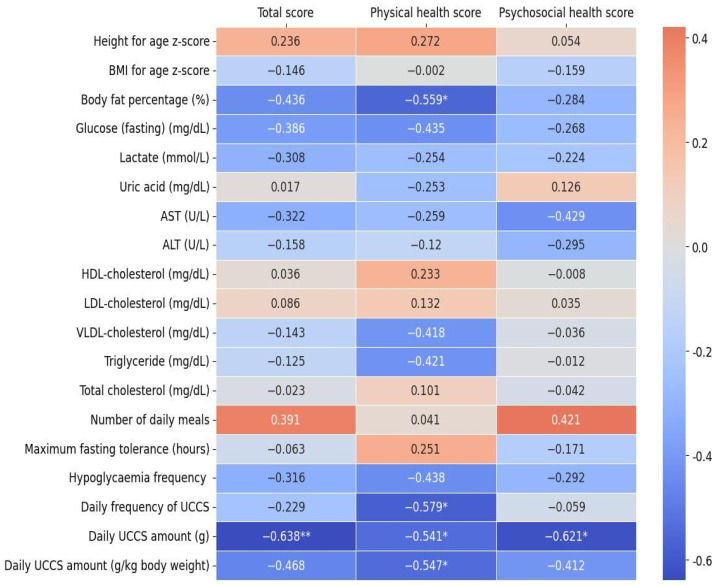
Exploratory correlations between children’s PedsQL scores and some parameters (Spearman correlation coefficients: * *p* < 0.05, ** *p* < 0.001). The analysis was adjusted for age and gender (Exact correlation coefficients, *p*-values, and sample sizes for each analysis are provided in Supplementary Table S2). ALT: Alanine aminotransferase, AST: Aspartate aminotransferase, BMI: Body mass index, HDL: High-density lipoprotein, LDL: Low-density lipoprotein, PedsQL: Pediatric Quality of Life Inventory, UCCS: Uncooked cornstarch, VLDL: Very low-density lipoprotein.

Exploratory correlation analyses between PedsQL scores and selected anthropometric, biochemical, and nutritional parameters are presented in Figure 1. Body composition and selected nutritional treatment burden indicators, including UCCS-related variables, were negatively correlated with some HRQoL domains (*p* < 0.05). A moderate negative correlation was also observed between the frequency of hypoglycemia and the physical health score, although this did not reach statistical significance (*p* = 0.053).

## 4. Discussion

Hepatic GSDs require lifelong dietary management to prevent hypoglycemia and maintain metabolic stability, including frequent intake of slow-digesting carbohydrates such as UCCS [4,5]. While clinical, biochemical, and anthropometric parameters are essential for monitoring metabolic control, they do not fully capture the physical, emotional, and social burden of the disease. Therefore, HRQoL assessment provides complementary information beyond traditional clinical indicators and is particularly relevant in chronic rare diseases such as hepatic GSD [30,31]. This study aimed to evaluate the HRQoL of children with hepatic GSD from both children’s and parents’ perspectives, examine discrepancies between reports, and explore associations between selected clinical and nutritional variables and HRQoL. In the present study, children with hepatic GSD had significantly lower total HRQoL score and nearly all subscale scores than both their healthy peers and children with other chronic diseases, according to both self- and parent-proxy reports. These findings are consistent with previous studies showing impaired HRQoL in children with GSD compared with healthy controls [7,12]. In children with GSD, strict dietary requirements may interfere with school meals, social participation, and daily activities, which may contribute to difficulties in social and school functioning [7,32,33]. Additionally, frequent meals and higher energy intake may be associated with increased body fat and perceived limitations in physical functioning, which may negatively affect perceptions of physical health [1,32]. The continuous need to adapt to disease-related restrictions may also contribute to social isolation and emotional difficulties [32,33].

According to Sobhy et al. [12], children with GSD showed significantly lower quality of life scores than healthy controls in both mental and physical domains. Similarly, Storch et al. [7] reported lower physical, psychosocial, and social functioning, and total scores in children with GSD type I compared to healthy peers, with parents also reporting significantly lower scores [7]. In the study by Storch et al. [7], emotional and school functioning scores were comparable to those of healthy children, which differs from our findings. This difference could be partly related to the clinical heterogeneity of hepatic GSD, as subtypes may share overlapping metabolic management concerns, particularly impaired fasting tolerance and risk of hypoglycemia, while differing in organ involvement, long-term complications, metabolic instability, dietary treatment burden, and their potential impact on HRQoL [33,34,35]. Considering the available subtype-level information and the clinical heterogeneity of the study population, the analyses were limited to exploratory comparisons rather than subtype-specific evaluations. Since GSD type I is generally characterized by more pronounced fasting intolerance, higher hypoglycemia risk, and more intensive dietary management, patients with type I were compared with those with other hepatic GSD subtypes to provide a clinically relevant descriptive perspective (Supplementary Table S1). However, because the comparison group was heterogeneous and the sample size was limited, these findings should be viewed as exploratory rather than subtype-specific. Future multicenter studies with larger and more homogeneous subtype-specific samples are warranted to clarify the relationship between disease severity, clinical course, and HRQoL outcomes.

When PedsQL scores in the study sample were compared with reference data for children with other chronic diseases, scores were significantly lower in children with GSD in all domains except emotional and school functioning, according to both child self-reports and parent-proxy reports. Although this was a contextual comparison based on external reference data, this pattern may suggest that hepatic GSD is associated with HRQoL challenges that may extend beyond those observed in heterogeneous chronic disease groups. This may partly reflect the combined burden of living with a rare disease and managing an inherited metabolic disorder [36,37,38]. Low disease awareness, stigmatization, social isolation, limited support networks, and restricted treatment options may affect HRQoL in rare diseases, while lifelong dietary treatment, metabolic monitoring, risk of metabolic decompensation, neurodevelopmental or functional complications, and family burden may further contribute to HRQoL impairment [36,37,38]. Evidence from studies involving different inherited metabolic diseases, including phenylketonuria, organic acidemias, lysosomal storage disorders, and heterogeneous pediatric inborn errors of metabolism cohorts, is consistent with this perspective [10,39,40]. These studies have emphasized that HRQoL may be influenced by dietary adherence, access to healthcare, financial difficulties, developmental disabilities, caregiver burden, progressive multisystem involvement, and disease-related functional limitations [10,39,40]. Although hepatic GSD differs clinically from these disorders, the shared rarity-related and metabolic management burdens may help explain why HRQoL challenges in hepatic GSD can be notable even when compared with heterogeneous chronic disease groups. Therefore, evaluation of HRQoL in hepatic GSD should extend beyond disease-specific metabolic outcomes and include broader psychosocial, school-related, and family-centered aspects of disease burden. This supports the value of comprehensive HRQoL assessment as part of routine care in pediatric hepatic GSD [30].

The patient’s self-report is considered the gold standard for measuring perceived HRQoL [15,41]. However, in the pediatric population, parent-proxy reports are recognized as providing valuable complementary information about patients’ well-being [17,41]. Therefore, this study evaluated HRQoL from both perspectives. In the present study, parents reported significantly lower scores than their children in the subscales of psychosocial health, social functioning, and school functioning, and total scores. Similar to findings in other chronic diseases, this may indicate that parents tend to perceive greater difficulties than their children report [15,42]. Discrepancies between child and parent reports may be related to parents’ limited awareness of their children’s daily social and school environments, heightened concerns about disease management, and the psychosocial burden associated with long-term caregiving [15,16,17,42,43]. Conversely, due to their developmental characteristics, children may underestimate or normalize the challenges they experience, or find it difficult to express their feelings [7,17]. Hence, evaluating HRQoL from both self- and proxy-reports is essential to capture a more complete understanding of children’s well-being [17,31].

Agreement analyses further supported the relevance of considering both perspectives, as ICC analyses showed poor to moderate agreement between child and parent reports in the present study [42,44]. Similar self-proxy discrepancies have also been reported in other inherited metabolic diseases [40,45]. In a Turkish cohort of patients with organic acidemias, Ersak et al. [45] reported differences between self- and proxy-reported outcomes, supporting the relevance of evaluating both perspectives in metabolic genetic disorders. More recently, Konomura et al. [40] assessed pediatric patients with various inborn errors of metabolism using self- and proxy-reported HRQoL measures together with a caregiver burden instrument and reported poor self-proxy agreement, as well as an association between lower proxy-reported HRQoL and higher caregiver burden. Taken together, these studies suggest that parent-proxy reports may reflect not only the child’s health status and daily functioning, but also parental well-being, caregiving burden, and emotional responses to long-term disease management [40,45]. In the present study, the lower parent-proxy scores may therefore partly reflect caregiver-related concerns surrounding the management of hepatic GSD. However, because parental HRQoL and caregiver burden were not formally assessed, this explanation could not be directly examined. Future studies incorporating child self-reports, parent-proxy reports, and validated measures of caregiver burden and parental HRQoL would further strengthen the dual-perspective framework and provide a more comprehensive understanding of HRQoL and family-centered support needs in pediatric hepatic GSD [42,43].

To better understand whether selected clinical and nutritional characteristics were related to HRQoL, exploratory correlation analyses were performed. Considering the small and heterogeneous study population, these analyses should be viewed as hypothesis-generating rather than confirmatory. In these analyses, selected nutritional management-related variables, including UCCS-related variables, were correlated with lower scores in some HRQoL domains. However, UCCS requirements are closely linked to disease subtype, metabolic control needs, and overall treatment burden; therefore, these associations should not be interpreted as a direct effect of UCCS intake on HRQoL [1,2]. Rather, they may reflect the broader burden of managing hepatic GSD in daily life, including the need for frequent carbohydrate intake and structured dietary routines [7,46]. In patients who require frequent UCCS administration or overnight nutritional support to maintain metabolic stability, night-time feeding or nocturnal cornstarch administration may further contribute to treatment burden and poorer HRQoL through sleep disruption in both children and caregivers, particularly in younger children who generally have lower fasting tolerance, may require more frequent feeding, and are more dependent on caregiver-assisted overnight management [47,48].

In the present study, body fat percentage was negatively correlated with physical health scores, which may point to a possible relationship between body composition and perceived physical functioning; however, these findings should be considered with caution, given the exploratory nature of the analyses. A moderate negative association was also observed between hypoglycemia frequency and physical health scores, but this did not reach statistical significance. No severe hypoglycemic episodes requiring hospital-based intervention were reported, and the reported episodes were mild to moderate according to patient- and/or caregiver-reported information. However, because hypoglycemia frequency and severity were based on patient- and/or caregiver-reported information rather than continuous glucose monitoring (CGM) or standardized severity grading, recall bias cannot be excluded, and asymptomatic or nocturnal episodes may have been underestimated. This may partly explain the lack of a statistically significant association between reported hypoglycemia frequency and HRQoL scores. Previous studies have shown that CGM may detect asymptomatic and nocturnal hypoglycemia and provide useful parameters for assessing glucose management in hepatic GSD [49,50]. Therefore, future studies should incorporate CGM as a methodological approach to objectively assess hypoglycemia burden, nocturnal hypoglycemia, glycemic variability, and CGM-derived indicators such as the depth and duration of hypoglycemic episodes in relation to HRQoL [50]. Moreover, no significant associations were found between biochemical parameters and HRQoL, although these findings should also be interpreted cautiously. In this context, individualized nutritional management that considers metabolic stability, body composition, hypoglycemia, and treatment burden may be relevant when evaluating both clinical status and HRQoL [32,33]. Assessing HRQoL as part of routine clinical and nutritional follow-up may help capture not only clinical status but also the physical, psychosocial, and family-centered challenges faced by children with hepatic GSD. In routine follow-up, brief HRQoL screening may help identify children and families who require additional psychosocial support, school-related counseling, or individualized dietary guidance.

The current study has several limitations. First, HRQoL data were obtained from a relatively small number of patients across a wide age range who were followed at a single center, which limited the ability to comprehensively examine demographic and disease-related factors. The clinical diversity and uneven distribution of hepatic GSD subtypes also limited robust subtype-specific analyses and may have contributed to residual confounding related to disease severity. Although exploratory comparisons between patients with GSD type I and other subtypes were performed, no statistically significant differences were observed, and these findings should be interpreted in light of the limited statistical power and the heterogeneity of the comparison group (Supplementary Table S1). Accordingly, subtype-specific interpretations should be made with caution. Similarly, exploratory correlations should not be interpreted as evidence of causal relationships or subtype-specific effects. Another limitation is that HRQoL scores were compared with reference values derived from healthy children and pediatric patients with chronic diseases in the United States rather than a culturally matched Turkish reference population. Although the Turkish version of the PedsQL has been validated, comprehensive Turkish normative data covering all relevant age groups and comparable clinical reference groups were not available. Because HRQoL perceptions and parent–child reporting patterns may be influenced by cultural, social, and healthcare system-related factors, these comparisons should be approached with caution and used as a descriptive reference rather than as direct population-level comparisons. Nevertheless, these reference data provided a standardized basis for placing the findings within the broader PedsQL literature. Furthermore, several variables, including hypoglycemia frequency and severity, were based on patient- and/or caregiver-reported information, which may introduce subjective and recall bias. Future studies incorporating multiple informants, objective assessment methods such as CGM, and larger multicenter subtype-specific samples may provide a more comprehensive evaluation of HRQoL in this population. Despite these limitations, the present study provides clinically relevant preliminary evidence on child- and parent-reported HRQoL in a rare and understudied pediatric hepatic GSD population. By integrating validated HRQoL measures with clinical, biochemical, anthropometric, and nutritional data, the study offers a broader perspective on disease burden beyond metabolic control alone.

## 5. Conclusions

In the present study, lower HRQoL scores were observed in children with hepatic GSD from both child and parent perspectives, supporting the value of considering child- and parent-reported outcomes alongside routine clinical, biochemical, anthropometric, and nutritional monitoring. Exploratory findings indicated that nutritional management-related variables and body composition may be associated with some HRQoL domains; however, these associations should be interpreted cautiously and should not be considered causal. Given the rarity of hepatic GSD and the limited HRQoL data available in this population, the present study provides clinically relevant preliminary evidence on the child- and parent-reported burden of the disease. Integrating brief HRQoL assessment into routine clinical and nutritional follow-up may help identify unmet psychosocial, school-related, and dietary support needs and inform individualized, family-centered care in pediatric hepatic GSD. Larger multicenter studies with more homogeneous subtype-specific samples, culturally comparable reference or control groups, and objective glycemic assessment are needed to better clarify factors associated with HRQoL in this population.

## Figures and Tables

**Table 1:** General characteristics of the sample (*n* = 23).

	*n*	%
Patient’s characteristics		
Age (years) (Median, IQR)	13.00 (8.00–16.00)	
Gender		
Male	15	65.2
Female	8	34.8
GSD Type		
I	5	21.8
III	9	39.2
IV	1	4.3
VI	1	4.3
IX	6	26.1
Fanconi–Bickel syndrome	1	4.3
Use of uncooked cornstarch		
Yes	18	78.3
No	5	21.7
Hypoglycemia frequency		
None	16	69.6
Rare (≤1/month)	5	21.7
Frequent (≥1/week)	2	8.7
Parental consanguinity status		
Yes	18	78.3
No	5	21.7
Mother’s characteristics		
Age (years) (Median, IQR)	38.00 (32.00–43.00)	
Education level		
Primary school	6	26.1
Secondary school	9	39.1
High school and above	8	34.8

**Table 2:** Anthropometric, Biochemical, and Nutritional Characteristics of the Patients (*n* = 23).

Parameters	Median	IQR	Values Outside Biochemical Reference Range, *n* (%) *
Height for age z-score	−1.52	−2.14–−0.45	NA
BMI for age z-score	0.27	−0.31–1.99	NA
Body fat percentage (%) (*n* = 20)	26.20	24.15–35.98	NA
Glucose (fasting) (mg/dL)	84.00	73.00–96.00	3 (13.00)
Lactate (mmol/L)	2.40	1.70–4.10	17 (73.90)
Uric acid (mg/dL)	4.90	3.90–6.40	5 (21.70)
AST (U/L)	75.00	33.00–219.00	17 (73.90)
ALT (U/L)	103.00	34.00–211.00	18 (78.30)
HDL-cholesterol (mg/dL)	40.00	30.00–51.00	10 (43.50)
LDL-cholesterol (mg/dL)	135.00	105.00–162.00	12 (52.20)
VLDL-cholesterol (mg/dL)	39.00	23.00–81.00	9 (39.10)
Triglyceride (mg/dL)	196.00	117.00–406.00	16 (69.60)
Total cholesterol (mg/dL)	194.00	158.00–248.00	13 (56.50)
Number of daily meals	7.00	6.00–9.00	NA
Maximum fasting tolerance (hours)	7.00	4.00–10.00	NA
Daily frequency of UCCS (*n* = 18)	2.00	1.75–4.25	NA
Daily UCCS amount (g) (*n* = 18)	48.00	31.50–148.00	NA
Daily UCCS amount (g/kg) (*n* = 18)	2.09	0.90–3.67	NA

BMI: Body mass index, AST: Aspartate aminotransferase, ALT: Alanine aminotransferase, HDL: High-density lipoprotein, LDL: Low-density lipoprotein, NA: Not applicable, VLDL: Very low-density lipoprotein, UCCS: Uncooked cornstarch. * For biochemical parameters, values outside the reference range were identified according to the age- and sex-specific reference intervals provided by the clinical laboratory. Values below the reference range were reported for glucose and HDL-cholesterol, whereas values above the reference range were reported for all other biochemical parameters.

**Table 3:** Comparison of PedsQL child self-report and parent-proxy report scores with normative data.

	Study Sample				Norm Data of Chronically Ill Children ^1^					Norm Data of Healthy Children ^3^				
	*n*	M	SD	95% CI	*n*	M	SD	*p* ^2^	*d*	*n*	M	SD	*p* ^4^	*d*
Child self-report														
Total score	20	62.45	16.97	54.50–70.39	367	77.19	15.53	<0.001	0.95	401	83.00	14.79	<0.001	1.38
Physical health	20	59.53	20.04	50.16–68.91	366	77.36	20.36	<0.001	0.88	400	84.41	17.26	<0.001	1.43
Psychosocial health	20	64.00	18.08	55.54–72.46	367	77.10	15.84	0.004	0.82	399	82.38	15.51	<0.001	1.18
Emotional functioning	20	64.50	21.14	54.60–74.40	366	76.40	21.48	0.021	0.55	400	80.86	19.64	0.003	0.83
Social functioning	20	67.75	24.57	56.25–79.25	367	81.60	20.24	0.021	0.68	399	87.42	17.18	0.002	1.12
School functioning	20	59.75	17.66	51.49–68.01	362	73.43	19.57	0.003	0.70	386	78.63	20.53	<0.001	0.93
Parent-proxy report														
Total score	23	52.42	15.35	45.94–59.38	662	74.22	18.40	<0.001	1.19	717	87.61	12.33	<0.001	2.83
Physical health	23	53.40	16.98	47.13–62.24	653	73.28	27.02	<0.001	0.74	717	89.32	16.35	<0.001	2.19
Psychosocial health	23	50.46	15.35	44.16–59.00	661	74.80	18.16	<0.001	1.35	717	86.58	12.79	<0.001	2.81
Emotional functioning	23	53.04	14.90	45.37–60.13	661	73.05	23.27	<0.001	0.87	718	82.64	17.54	<0.001	1.70
Social functioning	23	53.91	22.31	42.70–64.80	657	79.77	21.91	<0.001	1.18	716	91.56	14.20	<0.001	2.60
School functioning	21	48.73	15.75	40.76–55.74	601	71.08	23.99	<0.001	0.94	611	85.47	17.61	<0.001	2.09

^1^ Norm data of chronically ill children [25], ^2^ Sample vs. norm data of chronically ill children (one-sample *t*-test), ^3^ Norm data of healthy children [25], ^4^ Sample vs. norm data of healthy children (one-sample *t*-test). PedsQL: Pediatric Quality of Life Inventory, M: Mean, SD: Standard deviation, CI: Confidence interval, d: Cohen’s d.

**Table 4:** The PedsQL scores of children and their parents.

PedsQL Scores	Child Self-Report		Parent-Proxy Report		*p* ^1^	r	ICC
	Median	IQR	Median	IQR			
Total score	63.04	48.10–77.72	51.09	42.39–63.89	0.025	0.47	0.339
Physical health	59.38	41.41–77.35	51.57	43.75–65.63	0.116	0.33	0.451
Psychosocial health	63.34	50.83–75.86	48.33	41.67–56.67	0.015	0.51	0.250
Emotional functioning	60.00	46.25–87.50	52.50	40.00–60.00	0.076	0.37	0.023
Social functioning	70.00	50.00–92.50	52.50	40.00–65.00	0.024	0.47	0.431
School functioning	60.00	46.25–75.00	47.50	42.50–56.67	0.025	0.49	0.269

^1^ Wilcoxon signed-rank test. ICC: Intra-class correlations, IQR: Interquartile range, PedsQL: Pediatric Quality of Life Inventory, r: Rank biserial correlation.

## Data Availability

The datasets used and/or analyzed during the current study are available from the corresponding author upon reasonable request.

## Supplementary Materials

The following supporting information can be downloaded at https://www.mdpi.com/article/10.3390/nu18132099/s1. Table S1: Exploratory comparison of child- and parent-reported HRQoL scores between GSD type I and other subtypes, Table S2: Exploratory correlations between children’s PedsQL scores and some parameters.

## Abbreviations

The following abbreviations are used in this manuscript:

ALT	Alanine aminotransferase
AST	Aspartate aminotransferase
BMI	Body mass index
CI	Confidence interval
CGM	Continuous glucose monitoring
D	Cohen’s d
GSD	Glycogen storage disease
HDL	High-density lipoprotein
HRQoL	Health-related quality of life
ICC	Intraclass correlation coefficient
IQR	Interquartile range
LDL	Low-density lipoprotein
M	Mean
PedsQL	Pediatric Quality of Life Inventory
r	Rank biserial correlation coefficient
SD	Standard deviation
UCCS	Uncooked cornstarch
VLDL	Very low-density lipoprotein
WHO	World Health Organization
