# Supplementary material for: Health-Related Quality of Life in Pediatric Hepatic Glycogen Storage Disease: A Dual-Perspective Study
Source: Nutrients. 2026 Jun 27;18(13):2099. doi: 10.3390/nu18132099 (PMC13364485; doi:10.3390/nu18132099)
Supplement: Supplementary file 1 [file nutrients-18-02099-s001.zip › nutrients-4363605-supplementary.pdf]

Supplementary Table S1. Exploratory comparison of child- and parent-reported HRQoL scores between GSD type I and other subtypes.

	GSD Type I			Other Hepatic GSD Types			p*
	n	Median	IQR	n	Median	IQR	
**Child self-report**							
Total score	5	53.26	43.48 – 66.30	15	48.91	47.29 – 62.50	0.162
Physical health	5	68.75	50.00 – 81.25	15	43.75	31.25 – 59.38	0.060
Psychosocial health	5	65.00	53.33 – 83.33	15	53.33	47.50 – 71.67	0.359
Emotional functioning	5	60.00	50.00 – 95.00	15	60.00	42.50 – 80.00	0.660
Social functioning	5	75.00	55.00 – 75.00	15	50.00	47.50 – 75.00	0.292
School functioning	5	60.00	45.00 – 80.00	15	50.00	40.00 – 72.50	0.510
**Parent-proxy report**							
Total score	5	64.13	54.35 – 80.43	18	44.57	34.79 – 57.07	0.233
Physical health	5	53.13	43.75 – 68.75	18	43.75	42.19 – 62.50	0.478
Psychosocial health	5	53.33	43.33 – 60.00	18	45.00	30.83 – 54.17	0.247
Emotional functioning	5	55.00	45.00 – 65.00	18	40.00	37.50 – 55.00	0.143
Social functioning	5	55.00	40.00 – 65.00	18	40.00	20.00 – 62.50	0.258
School functioning	5	50.00	45.00 – 60.00	16	45.00	32.50 – 52.50	0.317

PedsQL: Pediatric Quality of Life Inventory,

** p values were calculated using the Mann–Whitney U test for exploratory purposes only and should be interpreted cautiously due to the small sample size.*

Supplementary Table S2. Exploratory correlations between children's PedsQL scores and some parameters.

		Total score		Physical health score		Psychosocial health score	
	**n**	**r**	**p**	**r**	**p**	**r**	**p**
Height for age z-score	20	0.236	0.316	0.272	0.246	0.054	0.821
BMI for age z-score	20	-0.146	0.539	-0.002	0.995	-0.159	0.502
Body fat percentage (%)	20	-0.436	0.055	**-0.559**	**0.010**	-0.284	0.225
Glucose (fasting) (mg/dL)	20	-0.386	0.092	-0.435	0.056	-0.268	0.254
Lactate (mmol/L)	20	-0.308	0.186	-0.254	0.280	-0.224	0.342
Uric acid (mg/dL)	20	0.017	0.945	-0.253	0.283	0.126	0.596
AST (U/L)	20	-0.322	0.166	-0.259	0.271	-0.429	0.059
ALT (U/L)	20	-0.158	0.506	-0.120	0.613	-0.295	0.207
HDL-cholesterol (mg/dL)	20	0.036	0.880	0.233	0.323	-0.008	0.975
LDL-cholesterol (mg/dL)	20	0.086	0.719	0.132	0.578	0.035	0.885
VLDL-cholesterol (mg/dL)	20	-0.143	0.548	-0.418	0.067	-0.036	0.880
Triglyceride (mg/dL)	20	-0.125	0.600	-0.421	0.064	-0.012	0.960
Total cholesterol (mg/dL)	20	-0.023	0.925	0.101	0.673	-0.042	0.860
Number of daily meals	20	0.391	0.088	0.041	0.865	0.421	0.064
Maximum fasting tolerance (hours)	20	-0.063	0.791	0.251	0.286	-0.171	0.470
Hypoglycaemia frequency	20	-0.316	0.175	-0.438	0.053	-0.292	0.212
Daily frequency of UCCS	16	-0.229	0.393	**-0.579**	**0.019**	-0.059	0.829
Daily UCCS amount (g)	16	**-0.638**	**0.008**	**-0.541**	**0.030**	**-0.621**	**0.010**
Daily UCCS amount (g/kg body weight)	16	-0.468	0.068	**-0.547**	**0.028**	-0.412	0.113

The analysis was adjusted for age and gender. PedsQL: Pediatric Quality of Life Inventory, BMI: Body mass index, ALT: Alanine aminotransferase, AST: Aspartate aminotransferase, HDL: High-density lipoprotein, LDL: Low-density lipoprotein, VLDL: Very low-density lipoprotein, UCCS: Uncooked cornstarch.
